# Strategy for the Management of Diabetic Macular Edema: The European Vitreo-Retinal Society Macular Edema Study

**DOI:** 10.1155/2015/352487

**Published:** 2015-01-28

**Authors:** Ron Adelman, Aaron Parnes, Zofia Michalewska, Barbara Parolini, Claude Boscher, Didier Ducournau

**Affiliations:** ^1^Yale University School of Medicine, New Haven, CT, USA; ^2^Eyecare Medical Group, Portland, ME, USA; ^3^Ophthalmic Clinic “Jasne Blonia”, Lodz, Poland; ^4^Department of Ophthalmology, S. Anna Hospital, Brescia, Italy; ^5^American Hospital of Paris, Paris, France; ^6^EVRS, Nantes, France

## Abstract

*Objective*. To compare the efficacy of different therapies in the treatment of diabetic macular edema (DME). *Design*. Nonrandomized, multicenter clinical study. *Participants*. 86 retina specialists from 29 countries provided clinical information on 2,603 patients with macular edema including 870 patients with DME.* Methods*. Reported data included the type and number of treatment(s) performed, the pre- and posttreatment visual acuities, and other clinical findings. The results were analyzed by the French INSEE (National Institute of Statistics and Economic Studies). *Main Outcome Measures*. Mean change of visual acuity and mean number of treatments performed. *Results*. The change in visual acuity over time in response to each treatment was plotted in second order polynomial regression trend lines. Intravitreal triamcinolone monotherapy resulted in some improvement in vision. Treatment with threshold or subthreshold grid laser also resulted in minimal vision gain. Anti-VEGF therapy resulted in more significant visual improvement. Treatment with pars plana vitrectomy and internal limiting membrane (ILM) peeling alone resulted in an improvement in vision greater than that observed with anti-VEGF injection alone. In our DME study, treatment with vitrectomy and ILM peeling alone resulted in the better visual improvement compared to other therapies.

## 1. Introduction

Diabetic macular edema (DME) is the leading cause of visual impairment in diabetic patients and according to some data is the leading cause of blindness among working age individuals in industrialized countries. The 10-year incidence of DME in diabetics was reported to be 20–40% [1]. Given the increasing incidence of diabetes, DME may become a leading cause of vision loss requiring treatment by ophthalmologists. A meta-analysis extrapolated to the world diabetes population in 2010 estimated that approximately 93 million people may have some form of diabetic retinopathy (DR), and 28 million may have sight-threatening stages of DR [2].

The optimal treatment of DME is evolving. Focal and grid laser photocoagulation's place as the gold standard of therapy for DME, as established in the Early Treatment of Diabetic Retinopathy Study (ETDRS), is threatened but still has a role in therapy [3].

New therapies continue to be developed and proven with an ever-growing swath of literature detailing positive clinical results. Antivascular endothelial growth factor (VEGF) agents have emerged as effective treatments and published data supports this tectonic shift [4–10]. The RISE and RIDE studies confirmed that intravitreal ranibizumab injection was superior to sham treatment and approximately 40% of patients treated monthly gained over 15 letters [6]. The RESOLVE study suggested that ranibizumab treatment was superior to laser (7.8 ETDRS letters gained versus −1.7 ETDRS letters lost) [9]. Similar results were obtained in the BOLT study, when bevacizumab was administered (8 ETDRS letters gained versus −0.5 ETDRS letters lost in patients treated with laser) [8]. It must be considered that although the endophthalmitis rate was confirmed to be as low as 0.006% in the RIDE and RISE trials and 2% in the RESOLVE study, diabetic patients typically require multiple injections and are in general more susceptible to infections and endophthalmitis [6, 9].

The question of timing in combined ranibizumab and laser therapy was explored by the Diabetic Retinopathy Clinical Research Network (DRCR) [11]. Two-year data confirmed that ranibizumab with deferred laser photocoagulation (>24 weeks) gained 5.7 more ETDRS letters, when compared to laser with sham injection. The protocol also compared ranibizumab with prompt laser and triamcinolone with prompt laser but found them in some aspect inferior to ranibizumab with deferred laser. Patients with ranibizumab and deferred laser needed a mean of 11.4 injections over 24 months. DRCR suggested that the smaller number of total injections, as compared to other trials when ranibizumab was used as solo therapy, may have been due to the laser photocoagulation performed [11].

Other therapies, such as intravitreal corticosteroid injections or implants, subthreshold laser photocoagulation, pars plana vitrectomy with or without internal limiting membrane (ILM) peeling, and combined therapies have supported clinical research and are also widely used [12–18]. Despite a large amount of primary literature, direct comparisons examining the efficacy of surgical and medical treatments, or combination thereof, are inadequate.

Prospective clinical trials that do exist in the literature, for the most part, only compare two or three treatment modalities. Grid laser photocoagulation and anti-VEGF agents have been commonly compared in recent years as monotherapy or in combination with each other [19–24]. The majority of these studies actually favor intravitreal anti-VEGF therapy over laser. Direct comparisons between intravitreal triamcinolone and anti-VEGF agents have been made as well [25, 26]. Turning to a surgical option, when measured against grid laser photocoagulation and intravitreal triamcinolone injection in relatively small clinical trials, pars plana vitrectomy had the advantage [27, 28].

It is not surprising that conducting comprehensive comparative studies for the treatment of DME is time consuming and costly. Prospective, randomized studies with just a few clinical arms are difficult enough to organize. One can imagine that a clinical trial with 10 or more treatment groups encompassing all current therapeutic options, and the many combinations that could be made between them, would be very difficult and very expensive to conduct. Alternatives to such an implausible randomized study must be examined.

European Vitreo-Retinal Society (EVRS) is an organization of over 1,900 retina specialists founded in 2001, which previously conducted large trials examining the treatment of retinal detachments [29–32]. A clinical study was initiated and participating EVRS members were asked to record information regarding individual cases of macular edema and the treatments performed since 2008. A total of 86 retina specialists from 29 countries provided information on 2,603 cases of macular edema with at least 6-month follow-up. In this report we will discuss the treatment and results of those cases with macular edema specifically related to diabetes.

## 2. Methods

The EVRS Macular Edema Study was a nonrandomized, multicenter study in which the goal was to analyze the treatment of macular edema. The focus was on the results of varying treatments and treatment combinations for each etiology of the macular edema. This paper concentrates on cases of DME and their treatment outcomes.

The members of EVRS contributed to the study by reporting on individual cases of macular edema and their management from 2008 to 2011. A portal was created on the EVRS website where reporting questionnaires were available to be filled out for each patient treated. At the conclusion of the reporting period, the study organizers received complete data on 2,603 cases of macular edema from 86 retina specialists. Follow-up for these cases ranged from 6 months to 2 years. The results were analyzed independent of investigators by the French INSEE (National Institute of Statistics and Economic Studies).

Reported data for each case included the type and number of treatment(s) utilized, the pretreatment and posttreatment visual acuities, and the specific dates of treatments and visual assessments. Lens status was also recorded. Information regarding complications was reported including an increased or new cataract, increased intraocular pressure, retinal detachment, vitreous hemorrhage, choroidal detachment, and macular hole. Macular optical coherence tomography (OCT) measurements were not reported by the surgeons in this investigation. After having cleaned the database, the global working sheet was sent to each contributor, masking the name of the other contributors, so that cleaning accuracy could be agreed upon.

Considering that this study was performed in 29 countries, the regulations and institutional review board requirements were different in each location. Every participant was responsible to follow the rules and regulations of each individual country and institution. In addition, the EVRS Ethics and Study Design Committees have approved the design and ethical aspects of the study.

This method of reporting of cases of macular edema led to a few difficulties. The large number of etiologies causing macular edema was the first factor limiting the ability to achieve statistical significance. While information on 2,603 eyes with macular edema was reported; 870 of those cases were specifically associated with diabetes. A second, and more influential, factor affecting the number of cases needed to reach a statistically significant comparison was treatment complexity. The relatively large number of treatment options available for macular edema and the lack of standardization in the integration of these treatment regimens presented some challenge. Given these limitations, the statisticians decided to present the results as trend lines displaying improvement in visual acuity over time.

Regarding the analysis of collected data, the institute of statistics made two decisions. First, a second order polynomial regression trend line method was used, as opposed to a linear or third order polynomial regression trend line, because it better illustrates the effect of various treatments. A linear regression simply does not reflect what happens to vision following a single treatment in clinical practice nor does a third order regression where acuity would be depicted as fluctuating up and down in an unnatural and unexpected way. The presentation of visual acuity over time with a second order trend line allows one to analyze the effect of treatment on vision at specific intervals or according to pretreatment visual acuity. The second decision made was that, for each data point plotted to create the trend lines, a minimum of three cases would be needed for every follow-up interval. With this approach, only the averages of vision improvement with at least three cases would be included, and the impact of aberrant cases would be minimized.

Additional analysis was performed in order to display final visual improvement (LogMAR) according to pretreatment visual acuity. A trend line combining the results for all treatments of DME was compared to plotted results for individual treatments and their average pretreatment visual acuities.

Considering that this is not a randomized study, there is a risk of selection bias, with regard to the cases reported on by each physician. Based on recommendation by the institute of statistics, this risk was limited by always comparing the results obtained with different treatment modalities as opposed to presenting individual values of visual improvement at different time points along the trend lines. Furthermore, the large number of reported cases and participating physicians in each treatment group worked to reduce selection bias. The trend lines, therefore, can be used to classify the efficiency of each treatment and must be considered as indicators of comparative results. Usually trend lines are useful in comparing treatment groups. However, they are not a precise measure of exact number of lines of improvement in vision. Based on this presentation, a strategy for the treatment of DME could be proposed.

## 3. Results

The details regarding the treatment of 2,603 cases of macular edema were reported by 86 retina specialists in 29 countries. Of these reported cases, 2,159 comprised the four etiologies, which had numbers large enough to study. 870 had DME, 551 had epiretinal membranes, 380 had branch retinal vein occlusion, and 358 had central retinal vein occlusion. The focus of this paper is to compare different treatment options for DME. The baseline demographic patient data are displayed in Table 1.

### 3.1. Monotherapy

**Figure 1:**
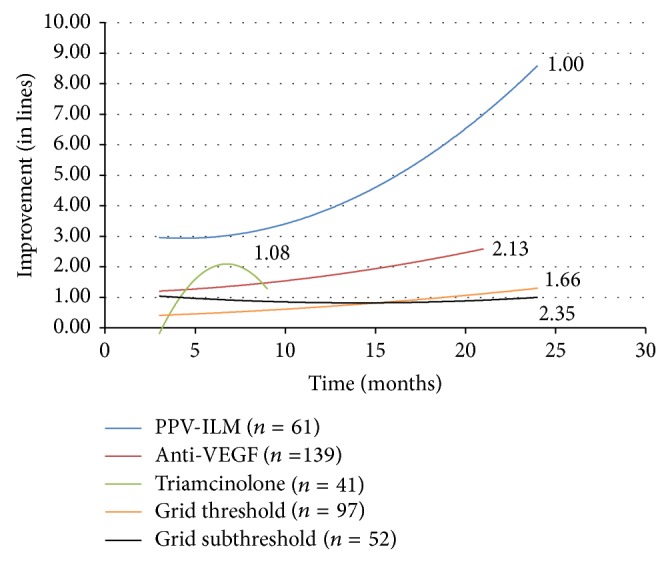
Change in visual acuity (in lines) with monotherapy. PPV-ILM = pars plana vitrectomy with internal limiting membrane peeling. VEGF = vascular endothelial growth factor.

Initial visual acuity in eyes in which pars plana vitrectomy was performed was lower than in all the other treatment groups (0.86 LogMAR versus 0.7 LogMAR in the anti-VEGF group, 0.69 LogMAR in the triamcinolone group, 0.39 LogMAR in threshold grid, and 0.36 LogMAR in the grid subthreshold group). Our first goal was to analyze the results of monotherapy in DME and to compare the efficacy of various treatment modalities. The evolution of visual acuity over time in response to each treatment is displayed as separate second order polynomial regression trend lines in Figure 1. The numbers adjacent to the trend lines indicate mean number of treatments for each therapeutic intervention.

Pars plana vitrectomy with ILM peeling was performed in 61 eyes and resulted in a trend line displaying marked visual recovery about 2-3 times higher when compared to anti-VEGF therapy alone. This result was superior to all other treatment groups. The improvement in visual acuity continued to increase between 12 and 24 months after surgery.

Monotherapy with anti-VEGF injections was performed in 139 eyes. Following anti-VEGF therapy alone, final visual improvement was over two times greater than the gains observed with either threshold (97 eyes) or subthreshold grid laser (52 eyes) (2.589 lines of improvement on the LogMAR chart at 21 months versus 1.326 lines and 0.995 lines at 24 months, resp.). The results achieved in subthreshold grid laser treated eyes were not statistically different from those obtained with threshold grid laser. Improvement remained constant throughout the follow-up period in both groups.

Triamcinolone injections were performed in 41 eyes. The trend line illustrating the response to treatment with intravitreal triamcinolone is truncated due to the fact that fewer than three cases were reported at each follow-up period past nine months. The fact that the therapy was discontinued after this period may be explained by the decrease of the visual acuity trend line.

An additional issue to be considered is the number of treatments per eye. Patients treated with vitrectomy and ILM peeling received a mean of 1 treatment per 24 months. Patients treated with anti-VEGF injections received a mean of 3 treatments per 24 months and a mean of 2 triamcinolone injections was performed during the first 9 months.

### 3.2. Combination Therapy

**Figure 2:**
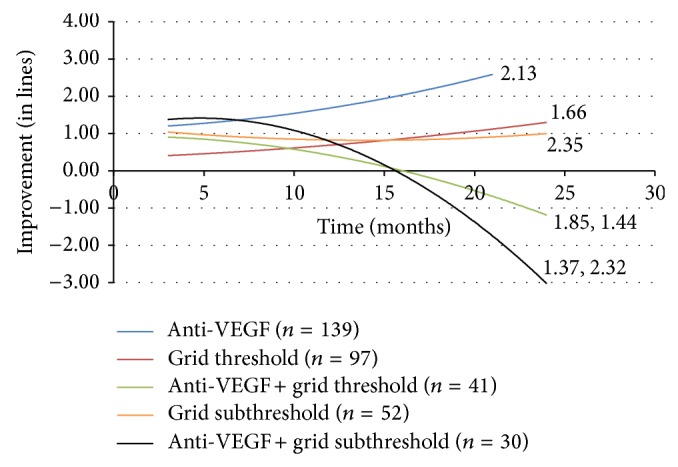
Change in visual acuity (in lines) with anti-VEGF combination therapy. VEGF = vascular endothelial growth factor.

The effect of the addition of grid laser photocoagulation to intravitreal anti-VEGF agent injection was compared to monotherapy (Figure 2). The improvement in visual acuity was similar to monotherapy at the 3 month mark. However, after 6 months the improvement in vision in the combination groups was lost and at 21 months vision worsening was observed with the addition of subthreshold grid laser to anti-VEGF therapy (loss of 3.017 lines of vision on the LogMAR chart). The addition of threshold grid laser to anti-VEGF injection resulted in a loss of vision at 24 months (loss of 1.199 lines).

**Figure 3:**
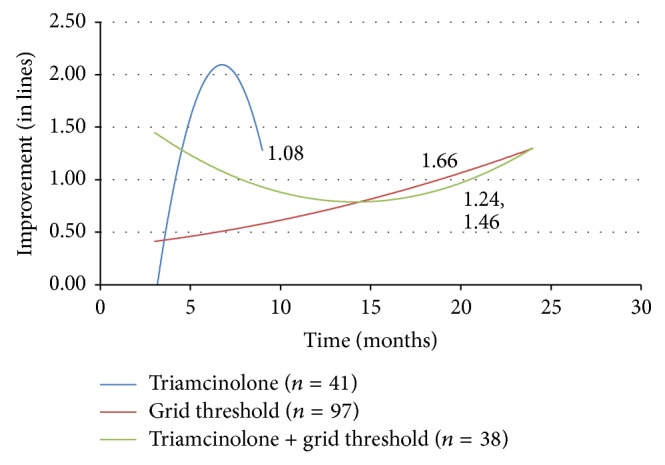
Change in visual acuity (in lines) with triamcinolone and threshold grid laser.

The combination of laser photocoagulation and triamcinolone was also investigated. At 3 months, improvement was observed in combination therapy when compared to either laser or steroid monotherapy. However, at 24 months both combination therapy (1.290 lines) and threshold grid laser alone led to a similar vision gain (Figure 3).

**Figure 4:**
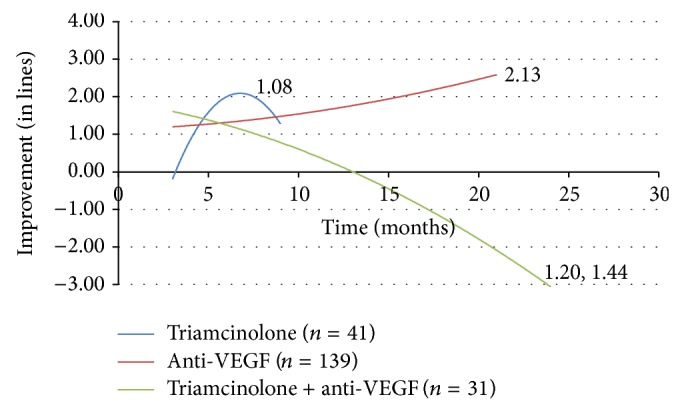
Change in visual acuity (in lines) with triamcinolone and anti-VEGF. VEGF = vascular endothelial growth factor.

**Figure 5:**
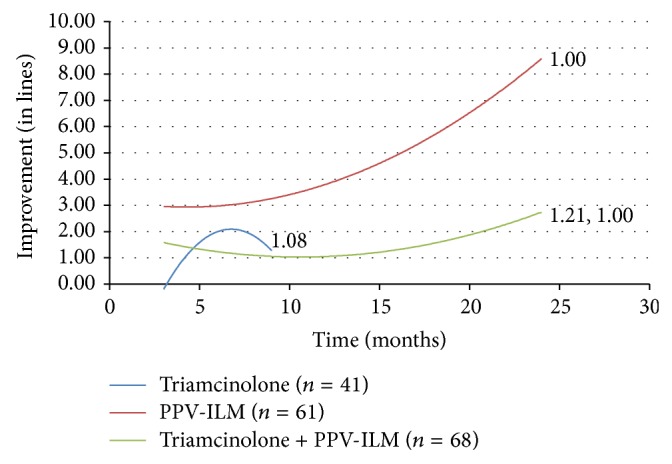
Change in visual acuity (in lines) with triamcinolone and PPV-ILM. PPV-ILM = pars plana vitrectomy with internal limiting membrane peeling.

The combination of intravitreal triamcinolone and anti-VEGF agents showed a temporary improvement in vision at 3 months when compared to monotherapy. However, combination therapy led to a negative result with an eventual vision loss at 24 months (loss of 3.052 lines) (Figure 4). The addition of triamcinolone injection to these cases receiving vitrectomy did not reach the level of visual improvement attained in those treated with ILM peeling alone (2.733 lines of gain) (Figure 5).

Visual improvement was then analyzed in terms of the percent of patients gaining three or six lines of vision. The last visual acuity reading available was compared to the recorded pretreatment visual acuity. The data of patients treated with either anti-VEGF injection or vitrectomy with ILM peeling alone were compared (Table 2). 31.3% of the anti-VEGF group and 55.2% of the vitrectomy group gained at least 3 lines of vision. This was a statistically significant difference. Vitrectomy with ILM peeling led to a significantly better result (*P* = 0.0031). 10.8% of those treated with anti-VEGF injection and 29.3% of patients receiving vitrectomy gained at least 6 lines of vision. Again, statistical significance was reached and ILM peeling had a more favorable outcome in this analysis.

**Figure 6:**
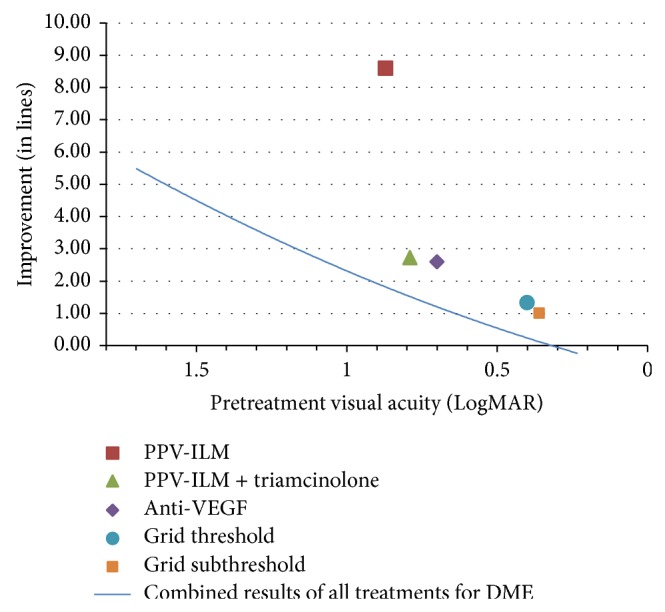
Final visual improvement (in lines) according to pretreatment visual acuity. PPV-ILM = pars plana vitrectomy with internal limiting membrane peeling. VEGF = vascular endothelial growth factor.

A final presentation of the data compared different treatment outcomes based on final visual improvement according to pretreatment visual acuity. In Figure 6, data were plotted as improvement in vision, in LogMAR, and against pretreatment visual acuity. A single second order regression trend line represents the combined results of all treatments for DME in this study, giving us a baseline. After analyzing all of the possible mono- and combination therapies for DME, the plotted data points represent the top five treatments in terms of vision improvement. Vitrectomy with ILM peeling showed the largest recovery in vision. This treatment was followed by, in order of descending amount of vision gain (LogMAR), vitrectomy with ILM peeling combined with triamcinolone (2.7328), anti-VEFG injection (2.5894), threshold grid laser (1.3256), and subthreshold grid laser (0.9953).

### 3.3. Complications

Intraocular pressure rise was observed most frequently in eyes treated with triamcinolone (17%). In the other treatment groups, intraocular pressure increased in about 3%.

Secondary cataract formation was most frequently observed in the pars plana vitrectomy group (14% versus 8% in the other treatment groups). Retinal detachment was observed in 1 case in the pars plana vitrectomy group, 1 case treated with anti-VEGF, and 1 case following grid laser photocoagulation.

## 4. Discussion

The choice of treatment when dealing with DME has been complicated in recent years with the emergence of new therapies, which allow for a large number of possible treatment schedules and combinations [3–28]. A prospective, randomized clinical trial could be done to address the efficacy of each treatment alone or in combination with another; however this sort of investigation would have many arms. Thus, the study would be almost prohibitively large, costly, and complex and would take years until results could be obtained. Here we present a nonrandomized, multicenter collaborative study to compare treatment effects among all available therapies.

When cases of DME treated with a single therapy were analyzed, monotherapy with vitrectomy and ILM peeling resulted in the largest improvement in vision. In comparison, treatment with pars plana vitrectomy and ILM peeling alone resulted in an improvement in vision two to three times greater than observed with anti-VEGF injection alone. Treatment with intravitreal anti-VEGF agents resulted in a better outcome than either type of grid laser, but vitrectomy still led to more vision improvement. Both methods of grid laser did not show overly positive results in improving visual acuity. It does appear that subthreshold grid laser has the same effect as threshold grid laser, which is consistent with the findings of other studies [27, 28]. These results suggest that if only a single therapy is to be considered to treat DME, vitrectomy with ILM peeling may be a good option for attaining visual acuity improvement over 24-month follow-up.

The more favorable outcomes observed with vitrectomy and ILM peeling alone versus other mainstream therapies certainly raise questions. Considering that surgery for DME is in many cases done as a last resort, why would this therapy alone result in significant visual improvement? A possible explanation could be that the majority of cases chosen for vitrectomy have already failed other treatments. Thus DME is chronic and photoreceptors are nonfunctional by the time that the operation is performed. In this situation, a better prognosis would be expected if a surgical option was opted for sooner.

The treatment of DME with anti-VEGF injection alone fared well with relatively good visual improvement; however the addition of any grid laser led to an overall negative outcome by the conclusion of the study. There are prior clinical trials supporting the assertion that the addition of grid laser to anti-VEGF therapy does not significantly improve outcomes [19, 21]. For example, the RESTORE study in 2011 did not show improvement with the addition of laser. Our results suggest that adding grid laser to a regimen of intravitreal anti-VEGF injections may not improve visual outcome. Prior studies have shown that outcomes for treatment of DME at 36 months with ranibizumab are maintained with frequent injections to optimally control edema and maximize vision. In our study the average number of intravitreal injections of anti-VEGF per patient was low which may reflect difficulty with frequent injections outside of randomized clinical trials. Also more extensive focal/grid laser therapy may have contributed in reduction of the number of injections [33].

Intravitreal triamcinolone is widely used to treat DME, especially in combination with other therapies. In this study, the efficacy of treating DME solely with triamcinolone injection is difficult to interpret given the truncated trend line with an insufficient number of cases to evaluate the effect on vision past nine months. This may reflect the general shift of therapy away from the use of triamcinolone injection to the use of anti-VEGF agents. There is sufficient data to comment on combination therapy with triamcinolone. While the addition of triamcinolone to threshold grid laser did not seem to help with vision improvement, the medication's addition to both anti-VEGF therapy and vitrectomy with ILM peeling actually led to worse results compared to cases where the steroid was not used. These results reflect the conclusions of clinical trials in the literature reporting no improvement with the addition of triamcinolone to anti-VEGF therapy or grid laser [22, 34–37]. The outcomes here suggest that triamcinolone injection may not be useful in augmenting treatment with grid laser, anti-VEGF injection, or ILM peeling.

A major issue that must be addressed is the fact that visual improvement should ideally be considered according to pretreatment vision level. It is less productive and less impactful to present the percentage of vision improvement greater than three lines where initial vision is not taken into account. ILM peeling was superior to anti-VEGF therapy in this comparison. However, this classical way of presenting data is not completely meaningful in this situation, since the improvement is dependent on pretreatment visual acuity. Certainly it is easier to achieve three lines of improvement if the initial vision is 20/400 than if it is 20/30. The true evaluation of results should relies on classification of the results according to pretreatment visual acuity. This was the impetus behind displaying final visual improvement for each therapy according to pretreatment visual acuity. According to this analysis, vitrectomy with ILM peeling resulted in better visual outcome for DME compared to other treatments.

The impact of vitrectomy on tractional diabetic macular edema is well known. Removal of the posterior hyaloid and release of traction result in an increase in tissue pressure and also a lowering of hydrostatic pressure gradient [38]. It is important to note that there is also evidence to support benefits of vitrectomy in nontractional edema. The oxygen level in the vitreous of diabetic patients is low [39]. There is evidence that demonstrate increased oxygen level in the vitreous cavity following vitrectomy [40]. Vitrectomy not only results in increased oxygen transport between the anterior and posterior segment, but also helps the removal of growth factors, such as vascular endothelial growth factor [41]. The net impact may result in improved macular edema. In addition, 3D spectral domain OCT now provides full-field macular images that can identify often overlooked extrafoveal traction that can contribute to DME that is labeled as nontractional by foveal features alone [42]. Also, PPV could result in better preservation of the ellipsoid line and parallelism and could therefore result in a better visual acuity.

Considering the success of ILM removal in improving retinal edema and vision in this study, a more detailed explanation regarding a possible mechanism of action is in order. Aside from the favorable changes with the removal of vitreous, peeling results in the removal of the ILM barrier as well as local glial proliferation including astrocytes, microglia, and the Müller cells endfeet. Prior investigations have shown that ILM peeling usually results in some Müller cell injury and stimulation of healing process [41]. Minor Müller cell trauma has been extensively studied in retinal detachments where it results in an upregulation of epidermal growth factor receptor (EGF-R). EGF-R regulates the injury response where stem cell proliferation compensates for the loss of neural cells. More importantly, EGF-R stimulates the filling of Müller cells with microfibrils of glial fibrillary acidic proteins (GFAPs) causing a vertical glial proliferation from the ILM to the external limiting membrane. This mechanism was described in the central nervous system, where, following trauma, radial glia are formed to repair and reconnect synapses [43, 44]. This has also been observed in the retina where, in the case of detachment, an increase of GFAPs in Müller cells attenuates hypoxic damage and neuronal loss is reduced [45–47]. Besides retinal detachment, these protective mechanisms have been shown to be linked to ischemic retinal injury [48, 49]. These results suggest that, as we perform vitrectomy and ILM peeling, anti-inflammatory agents like triamcinolone may not be helpful in reducing edema as they can decrease the repair of glial cells.

Significant weaknesses and limitations do exist for this sort of investigation. Considering this study was not randomized with specific treatment groups, the exact sequence and timing of single treatment types or combination therapies were not standardized. Different doses of triamcinolone, different techniques of grid laser application, and varying numbers of intravitreal anti-VEGF injections may have been used, possibly impacting the results. With regard to the formulation of the trend lines, even though several cases were available for each data point, for the most part fewer cases were available when plotting the data points two years out from starting treatment. This may weaken the validity of extrapolated final visual improvement. Trend lines are useful in comparing efficacy of therapeutic options. However, they may not be very precise in measuring exact number of lines of improvement in vision. Another issue is that not all diabetic macular edemas are created equal. It is possible that combination therapy, such as the addition of triamcinolone to another treatment or grid laser to anti-VEGF injection, was chosen in those cases deemed to be particularly severe or difficult. Thus, it is not that the combination is necessarily worse, but it is that the results may be affected due to a selection of patients with particularly advanced disease receiving that treatment.

It is extremely difficult to compare different studies due to varying inclusion criteria, treatment options, and initial visual acuities. Retrospective studies are generally of less statistical and clinical value than prospective trials. This weakness may be lessened by using a large number of investigated subjects, which can results in a more significant statistical analysis. Considering that this study shows outcomes similar to prior prospective randomized clinical trials with regard to anti-VEGF therapy with laser and triamcinolone with other treatment options, it is reasonable to infer that other outcomes from this investigation, such as the role of vitrectomy in the treatment of diabetic macular edema, may be later confirmed with subsequent prospective randomized trial results [8, 12, 15, 16].

In summary, this comparative study suggested that vitrectomy with ILM peeling may be a good option for the treatment of selected patients with DME. As for combination therapy, adding grid laser to a regimen of intravitreal anti-VEGF injections may not be helpful. Also, the addition of intravitreal triamcinolone injection to grid laser, anti-VEGF injection, or vitrectomy/ILM peeling may not improve final visual acuity. Additional prospective, randomized studies are needed to determine the optimal treatment of DME and confirm these results.

## Figures and Tables

**Table 1:** Baseline demographic patient data.

	Number of cases	Mean pretreatment Va LogMAR (Snellen)	Standard deviation	Mean number of treatments
Monotherapy				
Anti-VEGF only	139	0.7 (0.2)	0.41	2.13
Threshold grid	97	0.39 (0.41)	0.32	1.66
PPV-ILM	61	0.86 (0.14)	0.52	1.00
Subthreshold grid	52	0.36 (0.44)	0.25	2.35
Triamcinolone	41	0.69 (0.2)	0.40	1.08
Combination therapy				
Anti-VEGF (1) + threshold grid (2)	130	0.64 (0.23)	0.40	(1) 1.85 (2) 1.44
Anti-VEGF (1) + subthreshold grid (2)	30	0.61 (0.25)	0.37	(1) 1.37 (2) 2.32
Triamcinolone (1) + threshold grid (2)	38	0.64 (0.23)	0.46	(1) 1.24 (2) 1.46
Triamcinolone (1) + anti-VEGF (2)	31	0.62 (0.24)	0.39	(1) 1.20 (2) 1.44
Triamcinolone (1) + PPV-ILM (2)	68	0.79 (0.16)	0.39	(1) 1.21 (2) 1.00

VEGF = vascular endothelial growth factor.

PPV-ILM = pars plana vitrectomy with internal limiting membrane peeling.

**Table 2:** Final visual improvement for anti-VEGF injection and pars plana vitrectomy with ILM peeling monotherapy.

	Anti-VEGF (*n* = 102)	PPV-ILM (*n* = 58)	*P* value
≥3 line improvement	31.3%	55.2%	0.0031
≥6 line improvement	10.8%	29.3%	0.003

VEGF = vascular endothelial growth factor.

PPV-ILM = pars plana vitrectomy with internal limiting membrane peeling.
